# Supplementary material for: Brown adipose tissue is the key depot for glucose clearance in microbiota depleted mice
Source: Nat Commun. 2021 Aug 5;12:4725. doi: 10.1038/s41467-021-24659-8 (PMC8342435; doi:10.1038/s41467-021-24659-8)
Supplement: Supplementary file 1 — Supplementary Information [file 41467_2021_24659_MOESM1_ESM.pdf]

Supplementary information

Brown Adipose Tissue is the Key Depot for Glucose Clearance in Microbiota Depleted Mice

Min Li,[1,2,3,9] Li Li,[1,2,4,9] Baoguo Li,[1,2,5,9] Catherine Hambly,[3] Guanlin Wang,[1,2,3] Yingga Wu, [1,2,3] Zengguang Jin,[1] Anyongqi Wang,[1,2] Chaoqun Niu,[1] Christian Wolfrum,[6] & John R. Speakman[1,3,7,8]

Author information

[1]State Key Laboratory of Molecular Developmental Biology, Institute of Genetics and Developmental Biology, Chinese Academy of Sciences, Beijing, PR China.

[2]University of Chinese Academy of Sciences, Beijing, PR China.

[3]Institute of Biological and Environmental Sciences, University of Aberdeen, Aberdeen, Scotland, UK.

[4]Hypothalamic Research Center, Department of Internal Medicine, UT Southwestern Medical Center, Dallas, TX, USA.

[5]Department of Immunology, Weizmann Institute of Science, Rehovot, Israel.

[6]Institute of Food Nutrition and Health and Department of Health Sciences and Technology (ETH), Schwerzenbach, Switzerland

[7]CAS Center for Excellence in Animal Evolution and Genetics (CCEAEG), Beijing, PR China

[8]Shenzhen Key Laboratory of Metabolic Health, Center for Energy Metabolism and Reproduction, Shenzhen Institutes of Advanced Technology, Chinese Academy of Sciences, Shenzhen, PR China

[9]These authors contributed equally

email: j.speakman@siat.ac.cn

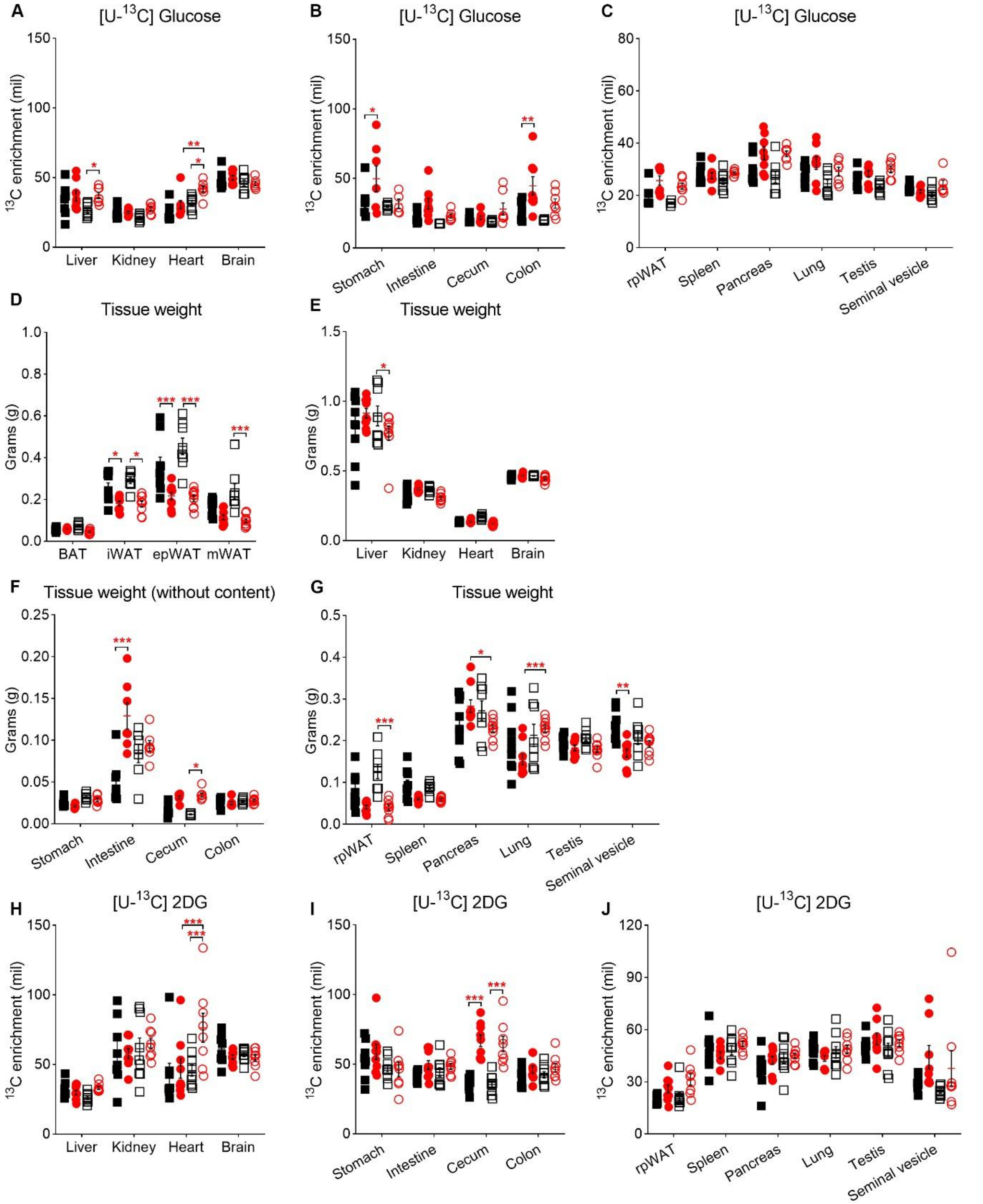

Supplementary Fig. 1 Gut microbiota depletion promotes glucose uptake in brown adipose tissue. Related to Figure 1.

(**a-c**) ^{13}C enrichment in liver (**a**) (Liver, P = 0.005; Heart:Ctrl:4°C vs ABX: 4°C, P = 0.017; Heart:ABX:22°C vs ABX: 4°C, P = 0.005), digestive organs (**b**) and other tissues (Stomach, P = 0.015; Colon, P = 0.004) (**c**) after intraperitoneal injection of [U-^{13}C] labelled glucose to mice at

22ºC or 48h acute cold exposure (Ctrl:22°C, n = 8; Ctrl:4°C, n = 8; ABX:22°C, n = 8; ABX:4°C, n = 7).

(**d-g**) Wet tissues mass in [U-^{13}C] glucose injected mice (Ctrl:22°C, n = 8; Ctrl:4°C, n = 8; ABX:22°C, n = 8; ABX:4°C, n = 7). (iWAT, 22°C, P = 0.048, 4°C, P = 0.001; epWAT, P < 0.001; mWAT, P < 0.001; Liver, P = 0.034; Intestine, P = 0.001; Cecum, P = 0.038; rpWAT, P < 0.001; Pancreas, P = 0.022; Lung, P < 0.001; Seminal vesicle, P = 0.003;)

(**h-j**) ^{13}C enrichment in in liver (**h**), digestive organs (**i**) and other tissues (**j**) after intraperitoneal injection of [U-^{13}C] labelled 2DG to mice at 22ºC or 48h acute cold exposure (Ctrl:22°C, n = 8; Ctrl:4°C, n = 9; ABX:22°C, n = 9; ABX:4°C, n = 8) (Heart, both shown P < 0.001; Cecum, both shown P < 0.001;).

All statistical analyses were performed by two-way ANOVA with Bonferroni's multiple comparisons. All results are given as mean ± SEM. Differences with P < 0.05 were considered to be significant. P < 0.05 (*), P < 0.01 (**), and P < 0.001 (***). iWAT, inguinal WAT. epWAT, epididymal WAT. mWAT, mesenteric WAT. rpWAT, retroperitoneal WAT.

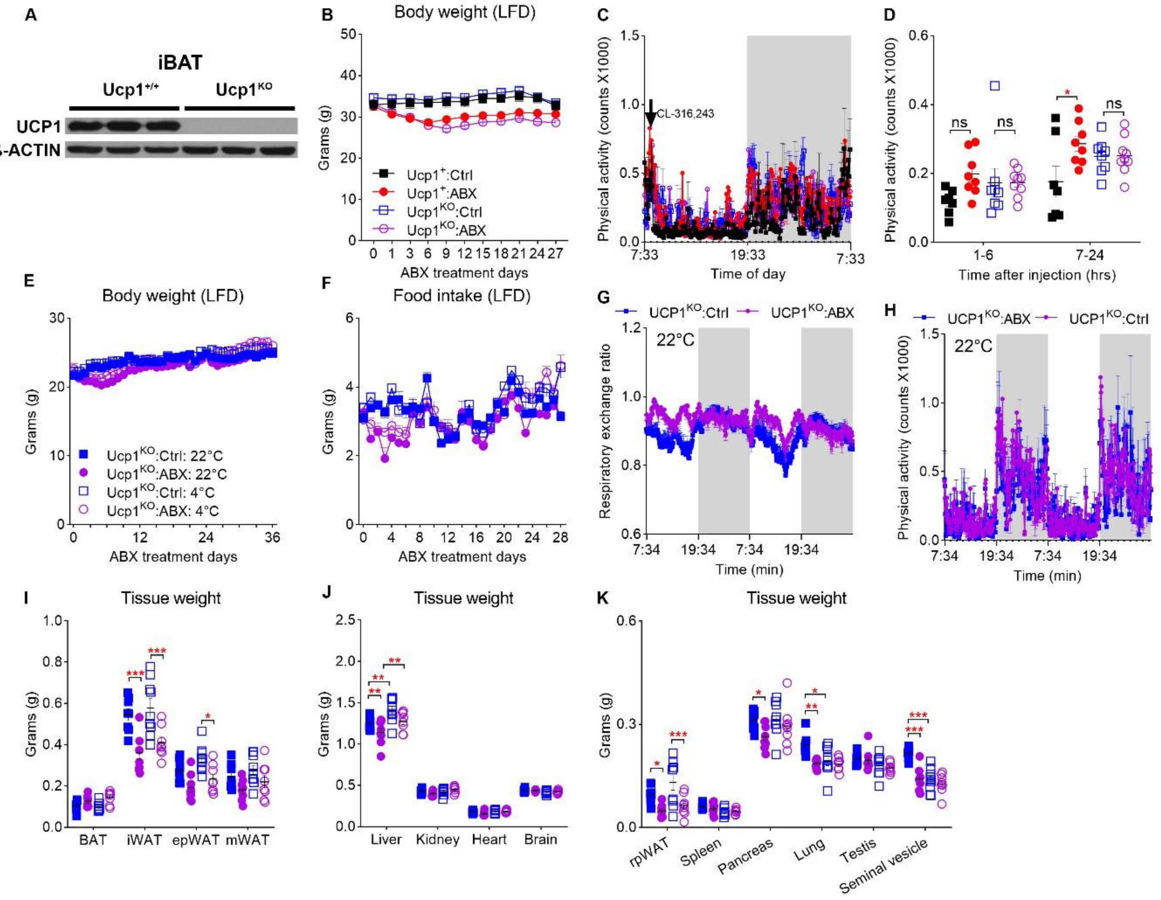

Supplementary Fig. 2 Gut microbiota is not required for UCP1-independent thermogenesis. Related to Figure 2.

(**a**) Validation of UCP1 deletion in Ucp1-KO mice using western blotting (n = 3 per group).

(**b**) Body mass curves (Ucp1^{+}: Ctrl, n = 7; Ucp1^{+}: ABX, n = 8; Ucp1^{KO}: Ctrl, n = 8; Ucp1^{KO}: ABX, n = 10).

(**c** and **d**) The curve (**c**) and average (**d**) of physical activity after CL-316246 injection (Ucp1^{+}: Ctrl, n = 7; Ucp1^{+}: ABX, n = 8; Ucp1^{KO}: Ctrl, n = 8; Ucp1^{KO}: ABX, n = 9) ($P = 0.030$).

(**e** and **f**) Daily body mass (**e**) and food intake (**f**) curves in Ucp1-KO mice (Ucp1^{KO}:Ctrl:22°C, n = 8; Ucp1^{KO}:Ctrl:4°C, n = 9; Ucp1^{KO}:ABX:22°C, n = 8; Ucp1^{KO}:ABX:4°C, n = 10)

(**g** and **h**) Respiratory exchange ratio (**g**) (n = 8 per group) and physical activity (**h**) (Ucp1^{KO}: Ctrl, n = 8; Ucp1^{KO}: ABX, n = 7) in ABX treated Ucp1-KO mice at 22°C.

(**i**-**k**) Wet tissues mass (Ucp1^{KO}:Ctrl:22°C, n = 8; Ucp1^{KO}:Ctrl:4°C, n = 9; Ucp1^{KO}:ABX:22°C, n = 8; Ucp1^{KO}:ABX:4°C, n = 8) (iWAT, both $P < 0.001$; epWAT, $P = 0.027$; Liver, $P = 0.004$; rpWAT, 22°C, $P = 0.021$, 4°C, $P < 0.001$; Pancreas, $P = 0.022$; Pancreas, $P = 0.022$; Lung, ABX, $P = 0.004$, 4°C, $P = 0.019$; Seminal vesicle, both $P < 0.001$). All statistical analyses were performed by two-way ANOVA with Bonferroni's multiple comparisons. All results are given as mean ± SEM. Differences with P < 0.05 were considered to be significant. $P < 0.05$ (*), $P < 0.01$ (**), and $P < 0.001$ (***).

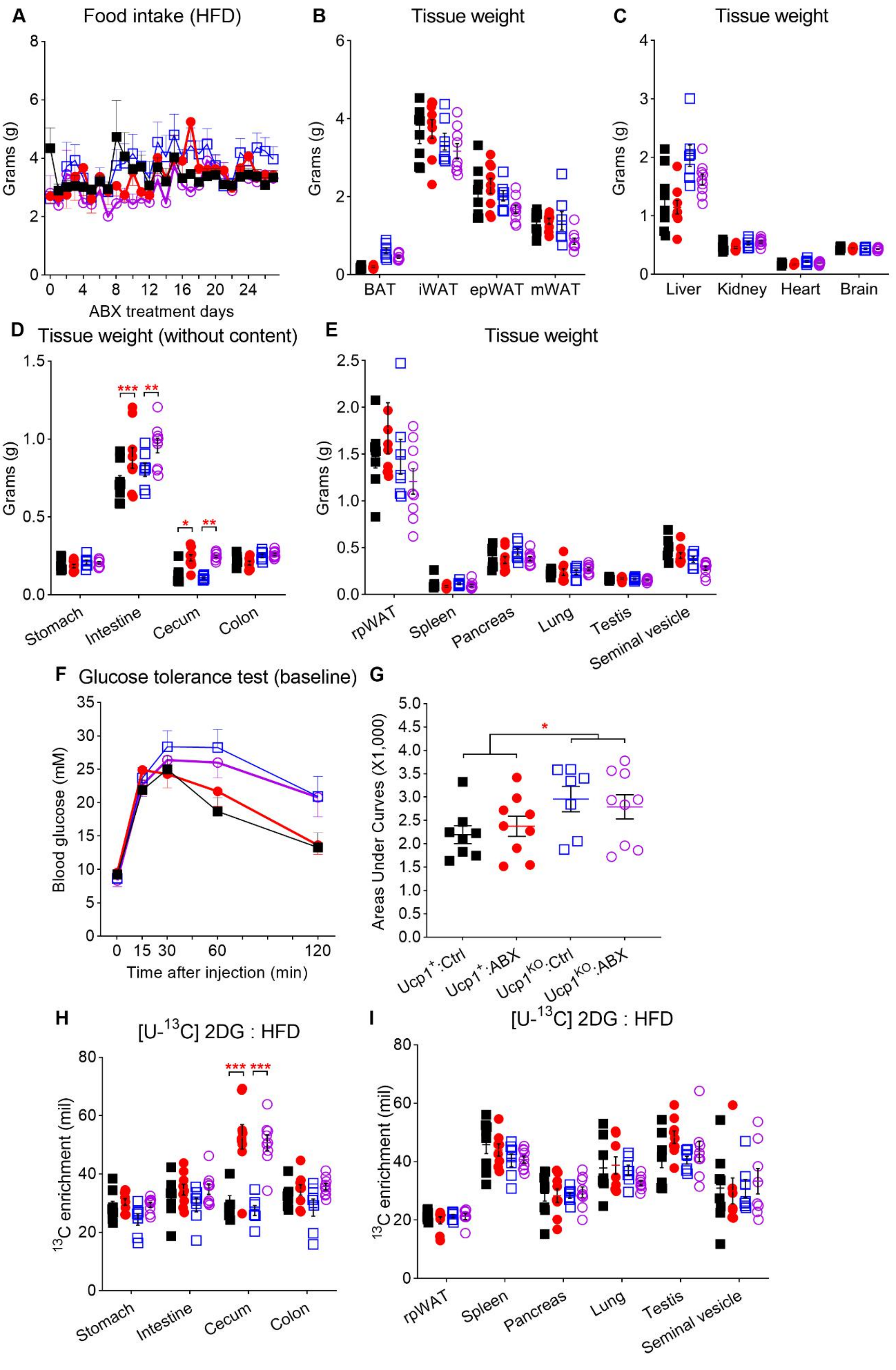

A
Food intake (HFD)
Grams (g)
ABX treatment days
B
Tissue weight
Grams (g)
BAT
iWAT
epWAT
mWAT
C
Tissue weight
Grams (g)
Liver
Kidney
Heart
Brain
D
Tissue weight (without content)
Grams (g)
Stomach
Intestine
Cecum
Colon
E
Tissue weight
Grams (g)
rpWAT
Spleen
Pancreas
Lung
Testis
Seminal vesicle
F
Glucose tolerance test (baseline)
Blood glucose (mM)
Time after injection (min)
G
Areas Under Curves (X1,000)
Ucp1+:Ctrl
Ucp1+:ABX
Ucp1KO:Ctrl
Ucp1KO:ABX
H
[U-13C] 2DG : HFD
13C enrichment (mil)
Stomach
Intestine
Cecum
Colon
I
[U-13C] 2DG : HFD
13C enrichment (mil)
rpWAT
Spleen
Pancreas
Lung
Testis
Seminal vesicle

Supplementary Fig. 3 UCP1 is dispensable for gut microbiota depletion induced glucose improvement. Related to Figure 3.

(**a**) Daily food intake curve of high fat diet (HFD) fed Ucp1-KO mice and its sibling control at Control or ABX condition ($Ucp1^{+}$: Ctrl, n = 8; $Ucp1^{+}$: ABX, n = 9; $Ucp1^{KO}$: Ctrl, n = 7; $Ucp1^{KO}$: ABX, n = 9).

(**b-e**) Wet tissues mass ($Ucp1^{+}$: Ctrl, n = 8; $Ucp1^{+}$: ABX, n = 9; $Ucp1^{KO}$: Ctrl, n = 7; $Ucp1^{KO}$: ABX, n = 9) (Intestine, $Ucp1^{+}$, $P < 0.001$; $Ucp1^{KO}$, $P = 0.002$; Cecum, $Ucp1^{+}$, $P = 0.035$; $Ucp1^{KO}$, $P = 0.007$).

(**f** and **g**) Intraperitoneal glucose tolerance test (GTT) and total glucose area under the curves (AUC) before ABX treatment ($Ucp1^{+}$: Ctrl, n = 8; $Ucp1^{+}$: ABX, n = 9; $Ucp1^{KO}$: Ctrl, n = 7; $Ucp1^{KO}$: ABX, n = 9).

(**h** and **i**) 2DG enrichment in digestive organs (**h**) (Cecum, both $P < 0.001$) and other tissues (**i**) after intraperitoneal injection of [U-^{13}C] labelled 2DG to mice at the fifth week of ABX ($Ucp1^{+}$: Ctrl, n = 8; $Ucp1^{+}$: ABX, n = 9; $Ucp1^{KO}$: Ctrl, n = 7; $Ucp1^{KO}$: ABX, n = 7).

All statistical analyses were performed by two-way ANOVA with Bonferroni's multiple comparisons. Data represent means ± SEM. Differences with $P < 0.05$ were considered to be significant. $P < 0.05$ (*), $P < 0.01$ (**), and $P < 0.001$ (***).

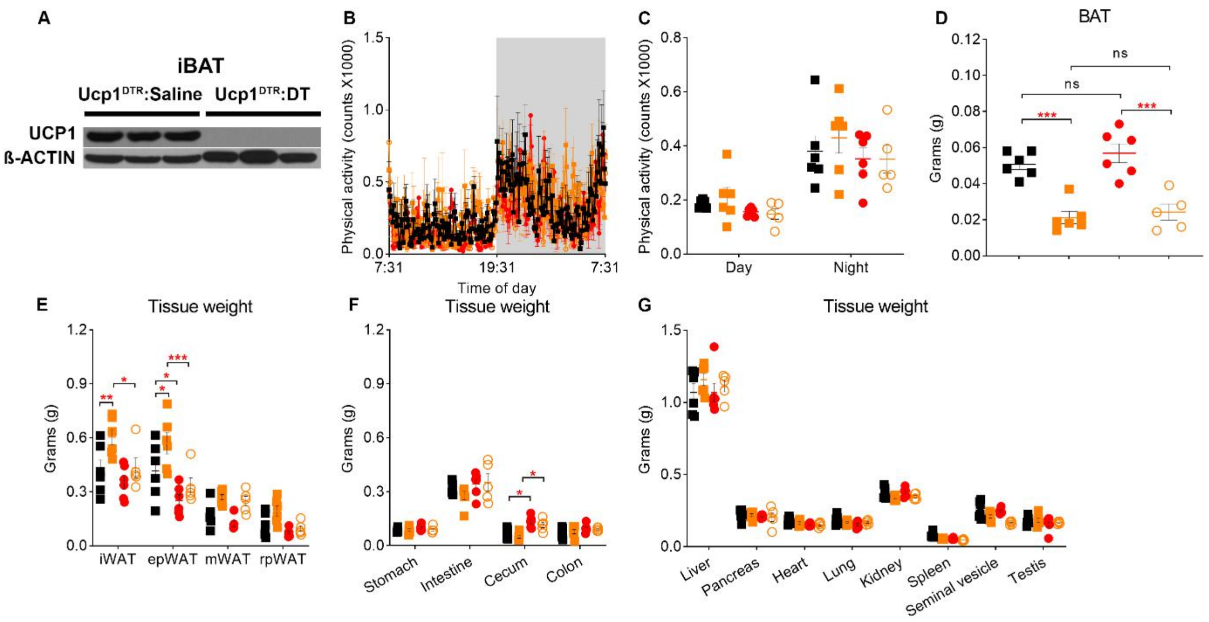

Supplementary Fig. 4 Deletion $Ucp1^{+}$ cells in lean mice blocked the gut microbiota depletion modulated glucose clearance improvement. Related to Figure 4.

10 weeks LFD-fed Ucp1-DTR mice were divided into 4 groups. Saline-Ctrl group (black square) received subcutaneous saline injections and drank autoclaved water; DT-Ctrl group (orange square) gave subcutaneous DT injection and drank autoclaved water; Saline-ABX group (red circle) received saline injections and ABX treatment; and DT-ABX group (orange open circle) gave subcutaneous DT injection and ABX treatment.

(**a**) Validation of UCP1 deletion in Ucp1-DTR mice using western blotting (n = 3 per group).

(**b** and **c**) The curve (**b**) and average (**c**) of physical activity ($Ucp1^{DTR}$:Saline:Ctrl, n = 6; $Ucp1^{DTR}$:DT:Ctrl, n = 6; $Ucp1^{DTR}$:Saline:ABX, n = 6; $Ucp1^{DTR}$:DT:ABX, n = 5).

(**d-g**) Wet tissues mass ($Ucp1^{DTR}$:Saline:Ctrl, n = 6; $Ucp1^{DTR}$:DT:Ctrl, n = 6; $Ucp1^{DTR}$:Saline:ABX, n = 6; $Ucp1^{DTR}$:DT:ABX, n = 5) (BAT, both $P < 0.001$; iWAT, $Ucp1^{DTR}$:Saline:Ctrl vs $Ucp1^{DTR}$:DT:Ctrl, $P = 0.007$; $Ucp1^{DTR}$:DT:Ctrl vs $Ucp1^{DTR}$:DT:ABX, $P = 0.020$; epWAT, $Ucp1^{DTR}$:Saline:Ctrl vs $Ucp1^{DTR}$:DT:Ctrl, $P = 0.034$; $Ucp1^{DTR}$:Saline:Ctrl vs $Ucp1^{DTR}$:Saline:ABX, P = 0.019; $Ucp1^{DTR}$:DT:Ctrl vs $Ucp1^{DTR}$:DT:ABX, $P < 0.001$; Cecum, Saline, $P = 0.035$; DT, $P = 0.048$).

DT, diphtheria toxin. DTR, diphtheria toxin receptor. All statistical analyses were performed by two-way ANOVA with Bonferroni's multiple comparisons. All results are given as mean ± SEM. Differences with P < 0.05 were considered to be significant. $P < 0.05$ (*), $P < 0.01$ (**), and $P < 0.001$ (***).

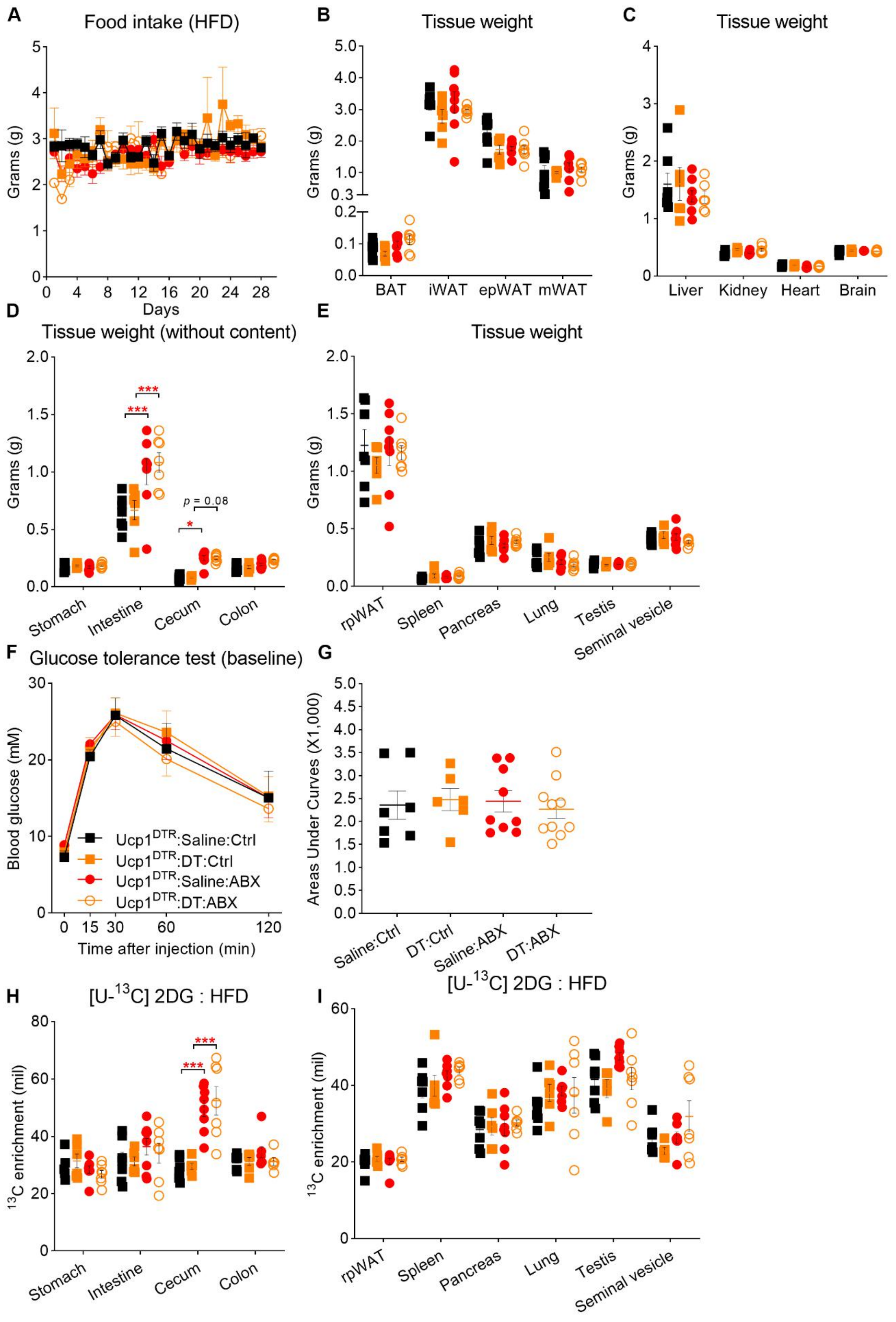
A
Food intake (HFD)
Grams (g)
Days
B
Tissue weight
Grams (g)
BAT
iWAT
epWAT
mWAT
C
Tissue weight
Grams (g)
Liver
Kidney
Heart
Brain
D
Tissue weight (without content)
Grams (g)
***
***
*
p = 0.08
Stomach
Intestine
Cecum
Colon
E
Tissue weight
Grams (g)
rpWAT
Spleen
Pancreas
Lung
Testis
Seminal vesicle
F
Glucose tolerance test (baseline)
Blood glucose (mM)
Ucp1DTR:Saline:Ctrl
Ucp1DTR:DT:Ctrl
Ucp1DTR:Saline:ABX
Ucp1DTR:DT:ABX
Time after injection (min)
G
Areas Under Curves (X1,000)
Saline:Ctrl
DT:Ctrl
Saline:ABX
DT:ABX
H
[U-13C] 2DG : HFD
13C enrichment (mil)
***
***
Stomach
Intestine
Cecum
Colon
I
[U-13C] 2DG : HFD
13C enrichment (mil)
rpWAT
Spleen
Pancreas
Lung
Testis
Seminal vesicle

Supplementary Fig. 5 Ucp1^{+} cells are indispensable for gut microbiota depletion induced glucose improvement in obese mice. Related to Figure 5.

10 weeks HFD-fed Ucp1-DTR mice were divided into 4 groups. Saline-Ctrl group (black square) received subcutaneous saline injections and drank autoclaved water; DT-Ctrl group (orange square) gave subcutaneous DT injection and drank autoclaved water; Saline-ABX group (red circle) received saline injections and ABX treatment; and DT-ABX group (orange open circle) gave subcutaneous DT injection and ABX treatment.

(**a**) Daily food intake curve of 4 different treated HFD fed Ucp1-DTR mice (Ucp1^{DTR}:Saline:Ctrl, n = 7; Ucp1^{DTR}:DT:Ctrl, n = 6; Ucp1^{DTR}:Saline:ABX, n = 9; Ucp1^{DTR}:DT:ABX, n = 9).

(**b**-**e**) Wet tissues mass (Ucp1^{DTR}:Saline:Ctrl, n = 7; Ucp1^{DTR}:DT:Ctrl, n = 6; Ucp1^{DTR}:Saline:ABX, n = 8; Ucp1^{DTR}:DT:ABX, n = 7) (Intestine: both $P < 0.001$; Cecum: Saline, $P = 0.047$;).

(**f** and **g**) Intraperitoneal glucose tolerance test (GTT) and total glucose area under the curves (AUC) before ABX treatment (Ucp1^{DTR}:Saline:Ctrl, n = 7; Ucp1^{DTR}:DT:Ctrl, n = 6; Ucp1^{DTR}:Saline:ABX, n = 10; Ucp1^{DTR}:DT:ABX, n = 10).

(**h** and **i**) 2DG enrichment in digestive organs (**h**) and other tissues (**i**) after intraperitoneal injection of [U-^{13}C] labelled 2DG to mice at the fifth week of ABX (Ucp1^{DTR}:Saline:Ctrl, n = 7; Ucp1^{DTR}:DT:Ctrl, n = 6; Ucp1^{DTR}:Saline:ABX, n = 8; Ucp1^{DTR}:DT:ABX, n = 7) (Cecum: both $P < 0.001$;)

DT, diphtheria toxin. DTR, diphtheria toxin receptor. All statistical analyses were performed by two-way ANOVA with Bonferroni's multiple comparisons. Data represent means ± SEM. Differences with P < 0.05 were considered to be significant. $P < 0.05$ (*), $P < 0.01$ (**), and $P < 0.001$ (***).
