# Supplementary material for: Brown adipose tissue is the key depot for glucose clearance in microbiota depleted mice
Source: Nat Commun. 2021 Aug 5;12:4725. doi: 10.1038/s41467-021-24659-8 (PMC8342435; doi:10.1038/s41467-021-24659-8)
Supplement: Supplementary file 3 — Reporting Summary [file 41467_2021_24659_MOESM3_ESM.pdf]

nature research

Corresponding author(s): NCOMMS-21-00799A-Z

Last updated by author(s): Jun 1, 2021

Reporting Summary

Nature Research wishes to improve the reproducibility of the work that we publish. This form provides structure for consistency and transparency in reporting. For further information on Nature Research policies, see our Editorial Policies and the Editorial Policy Checklist.

Statistics

For all statistical analyses, confirm that the following items are present in the figure legend, table legend, main text, or Methods section.

n/a	Confirmed	
☐	☒	The exact sample size (n) for each experimental group/condition, given as a discrete number and unit of measurement
☐	☒	A statement on whether measurements were taken from distinct samples or whether the same sample was measured repeatedly
☐	☒	The statistical test(s) used AND whether they are one- or two-sided<br>*Only common tests should be described solely by name; describe more complex techniques in the Methods section.*
☐	☒	A description of all covariates tested
☐	☒	A description of any assumptions or corrections, such as tests of normality and adjustment for multiple comparisons
☐	☒	A full description of the statistical parameters including central tendency (e.g. means) or other basic estimates (e.g. regression coefficient) AND variation (e.g. standard deviation) or associated estimates of uncertainty (e.g. confidence intervals)
☐	☒	For null hypothesis testing, the test statistic (e.g. F, t, r) with confidence intervals, effect sizes, degrees of freedom and P value noted<br>*Give P values as exact values whenever suitable.*
☒	☐	For Bayesian analysis, information on the choice of priors and Markov chain Monte Carlo settings
☒	☐	For hierarchical and complex designs, identification of the appropriate level for tests and full reporting of outcomes
☒	☐	Estimates of effect sizes (e.g. Cohen's d, Pearson's r), indicating how they were calculated

Our web collection on statistics for biologists contains articles on many of the points above.

Software and code

Policy information about availability of computer code

Data collection	Indirect calorimetry system - TSE PhenoMaster;<br>EchoMRI
Data analysis	GraphPad version 8<br>Minitab 16<br>ImageJ 1.50i<br>R 4.03

For manuscripts utilizing custom algorithms or software that are central to the research but not yet described in published literature, software must be made available to editors and reviewers. We strongly encourage code deposition in a community repository (e.g. GitHub). See the Nature Research guidelines for submitting code & software for further information.

Data

Policy information about availability of data

All manuscripts must include a data availability statement. This statement should provide the following information, where applicable:

- Accession codes, unique identifiers, or web links for publicly available datasets
- A list of figures that have associated raw data
- A description of any restrictions on data availability

All C13 raw data are provided in the supplementary excel file.

Field-specific reporting

Please select the one below that is the best fit for your research. If you are not sure, read the appropriate sections before making your selection.

☒ Life sciences ☐ Behavioural & social sciences ☐ Ecological, evolutionary & environmental sciences

For a reference copy of the document with all sections, see nature.com/documents/nr-reporting-summary-flat.pdf

Life sciences study design

All studies must disclose on these points even when the disclosure is negative.

Sample size	The sample sizes (6-9 mice per group) were determined on the basis of previous experiments using similar methods (doi: 10.1016/j.celrep.2019.02.015).
Data exclusions	No data were excluded from the analyses. Because the C13 samples were collected in Beijing China and the isotope enrichments were analyzed in Aberdeen UK, there is a small number of C13 isotope samples missed during the transportations (76/2772).
Replication	All the experiments such as body weight, body composition, food intake, GTT and oxygen consumption are the results of one biological replicate from 2-3 cohorts that were performed. Exact details between experiments were not identical but the trends were always the same and the differences between replicates not substantive.<br>The only experiment without biological replication was the C13 experiment but all C13 samples in 22 tissues were measured in two technical replication. To verify the reproducibility in C13 experiment, we observed a similar trends in 19 tissues between C13-glucose and C13-2DG tracer experiment.
Randomization	All experiments were randomly allocated to groups.
Blinding	Because of the nature of the treatments it was not possible for the observers to be blind to the experimental groups when the live experiments were performed - ie the oxygen consumption and body temperature experiments. That is it is impossible to blind an observer working on mice at 4 oC from the fact those mice are at 4 oC, since the person needs to enter the cold room to weigh them etc. Once samples were collected however all analyses were performed blind to the treatment allocations.

Reporting for specific materials, systems and methods

We require information from authors about some types of materials, experimental systems and methods used in many studies. Here, indicate whether each material, system or method listed is relevant to your study. If you are not sure if a list item applies to your research, read the appropriate section before selecting a response.

Materials & experimental systems

n/a	Involved in the study	
☐	☒	Antibodies
☒	☐	Eukaryotic cell lines
☒	☐	Palaeontology and archaeology
☐	☒	Animals and other organisms
☒	☐	Human research participants
☒	☐	Clinical data
☒	☐	Dual use research of concern

Methods

n/a	Involved in the study	
☒	☐	ChIP-seq
☒	☐	Flow cytometry
☒	☐	MRI-based neuroimaging

Antibodies

Antibodies used	UCP1 (Abcam, Cat# ab10983, 1:3,000 diluted)<br>β-actin (ZSGB-Bio, Cat# TA-09, 1:5,000 diluted)<br>Goat anti-Mouse IgG HRPantibody (ZSGB-Bio, Cat#ZB-2305, 1:5,000 diluted)<br>Goat anti-Rabbit IgG HRP antibody (ZSGB-Bio, Cat#ZB-2301, 1:5,000 diluted)
Validation	UCP1 antibody was validated in Ucp1-KO mice (Sup Fig 2a) and a previous study (doi.org/10.1038/s42255-019-0101-4).<br>β-actin antibody was validated by vendor and our previous publications (doi: 10.1016/j.celrep.2019.02.015).<br>HRP secondary antibodies were validated by vendor and our previous publications (doi: 10.1016/j.celrep.2019.02.015).

Animals and other organisms

Policy information about studies involving animals; ARRIVE guidelines recommended for reporting animal research

Laboratory animals	Mouse: C57Bl/6N-SPF; Charles River Laboratories; Cat# CRL_27, RRID:IMSR_CRL:27. In HFD experiments, mice were fed with high-fat

diet starting at 9-week old for 10 weeks, then were treated with ABX for about 4-5 weeks.
Mouse: UCP1-KO; Shanghai model organisms; Cat# NM-KO-190668, RRID:IMSR_NM-KO-190668
Mouse: UCP1-DTR-eGFP; Christian Wolfrum; (Rosenwald et al., 2013)
All mouse experiments were performed in male mice. Mice were housed in specific-pathogen-free facility (SPF) kept at 23 ± 1oC and 50% humidity with a dark-light cycle of 12h:12h (lights on at 07:30) and fed ad libitum with a standard chow diet (20% kcal Protein, 70% kcal Carbohydrate and 10% kcal Fat, #D12450B, Research Diets, New Brunswick, NJ) in all LFD experiments or with a high-fat diet (20% kcal Protein, 20% kcal Carbohydrate and 60% kcal Fat, #D12492, Research Diets, New Brunswick, NJ) in all HFD experiments. In HFD experiments, mice were fed with high-fat diet starting at 9-week old for 10 weeks, then were treated with ABX for about 4-5 weeks.

Wild animals	The study did not involve wild animals.
Field-collected samples	The study did not involve samples collected from the field.
Ethics oversight	All animal procedures were approved by the Institute of Genetics and Developmental Biology Chinese Academy of Sciences (IGDB-CAS) Institutional Animal Care and Use Committee (IACUC).

Note that full information on the approval of the study protocol must also be provided in the manuscript.
